# Exosomes derived from miR‐375‐overexpressing human adipose mesenchymal stem cells promote bone regeneration

**DOI:** 10.1111/cpr.12669

**Published:** 2019-08-05

**Authors:** Si Chen, Yiman Tang, Yunsong Liu, Ping Zhang, Longwei Lv, Xiao Zhang, Lingfei Jia, Yongsheng Zhou

**Affiliations:** ^1^ Department of Prosthodontics Peking University School and Hospital of Stomatology Beijing China; ^2^ 4th Division Peking University Hospital of Stomatology Beijing China; ^3^ National Engineering Lab for Digital and Material Technology of Stomatology Peking University School and Hospital of Stomatology Beijing China; ^4^ National Clinical Research Center for Oral Diseases Beijing China; ^5^ Central Laboratory Peking University School and Hospital of Stomatology Beijing China

**Keywords:** adipose mesenchymal stem cells, bone regeneration, exosomes, gene delivery, miR‐375

## Abstract

**Objectives:**

The present study aimed to investigate whether exosomes derived from miR‐375‐overexpressing human adipose mesenchymal stem cells (hASCs) could enhance bone regeneration.

**Materials and Methods:**

Exosomes enriched with miR‐375 (Exo [miR‐375]) were generated from hASCs stably overexpressing miR‐375 after lentiviral transfection and identified with transmission electron microscopy, nanosight and western blotting. The construction efficiency of Exo (miR‐375) was evaluated with qRT‐PCR and incubated with human bone marrow mesenchymal stem cells (hBMSCs) to optimize the effective dosage. Then, the osteogenic capability of Exo (miR‐375) was investigated with ALP and ARS assays. Furthermore, dual‐luciferase reporter assay and western blotting were conducted to reveal the underlying mechanism of miR‐375 in osteogenic regulation. Finally, Exo (miR‐375) were embedded with hydrogel and applied to a rat model of calvarial defect, and μ‐CT analysis and histological examination were conducted to evaluate the therapeutic effects of Exo (miR‐375) in bone regeneration.

**Results:**

miR‐375 could be enriched in exosomes by overexpressing in the parent cells. Administration of Exo (miR‐375) at 50 μg/mL improved the osteogenic differentiation of hBMSCs. With miR‐375 absorbed by hBMSCs, *insulin‐like growth factor binding protein 3* (*IGFBP3*) was inhibited by binding to its 3′UTR, and recombinant IGFBP3 protein reduced the osteogenic effects triggered by Exo (miR‐375). After incorporated with hydrogel, Exo (miR‐375) displayed a slow and controlled release, and further in vivo analysis demonstrated that Exo (miR‐375) enhanced the bone regenerative capacity in a rat model of calvarial defect.

**Conclusions:**

Taken together, our study demonstrated that exosomes derived from miR‐375‐overexpressing hASCs promoted bone regeneration.

## 1 INTRODUCTION

Mesenchymal stem cells (MSCs) are broadly utilized in bone tissue engineering owing to their ability of multipotential differentiation. Recently, mounting evidences have indicated that transplanted MSCs exert their therapeutic action by paracrine secretion of cytokines rather than through direct cell replacement.^1^, ^2^ As a class of extracellular vesicles, exosomes play a considerable role in paracrine regulation. Encapsulated with a lipid bilayer, exosomes can protect its contents from degradation and transport a variety of small biomolecules including mRNAs, miRNAs, non‐coding RNAs and proteins to surrounding cells.^3^, ^4^ As natural vesicles of gene delivery, MSC‐derived exosomes exhibit a broad range of therapeutic effects, which were previously attributed to MSCs, such as tissue repair, immunological regulation and inflammatory control.^5^, ^6^, ^7^ Moreover, recent studies have revealed MSC‐derived exosomes were able to regulate osteogenic differentiation, promote bone regeneration and ameliorate osteopenia in vivo.^8^, ^9^, ^10^

Despite their great potential in therapeutic delivery, MSC‐derived exosomes have shown limited application in clinical studies because of many problems, and the low yield poses a major challenge to further applications.^11^, ^12^ Several strategies have been developed to facilitate the release of exosomes, including raising intracellular calcium concentration and serum starvation. However, such external treatments could run the risk of altering the contents and functionality of MSC‐derived exosomes.^13^ Another effective strategy to expand exosome production is to find high‐output MSC source. Lim SK et al demonstrated that transfer of the oncogene *c‐myc* was an available strategy to gain an abundant exosome production.^14^ However, transfer of *c‐myc* into healthy cells could increase the therapeutic risks due to the tumourigenic potential. Human adipose mesenchymal stem cells (hASCs) are an ideal MSC type for producing large quantities of exosomes, due to the advantages of rapid proliferation and wide distribution in the human body.^15^, ^16^ Our previous study confirmed the safety and effectiveness of hASC‐derived exosomes in the regeneration of critical‐sized calvarial defects when constructed with PLGA/pDA scaffolds.^17^ However, only exosomes secreted by osteogenically induced hASCs could exert osteoinductive effects, and exosomes secreted by hASCs without osteogenic induction had no significant osteoinductive effects, which would undoubtedly increase the risk of contamination during the prolonged cultivation process. Recent studies demonstrated great potential for exosomes to carry therapeutic genes.^18^, ^19^, ^20^ Thus, we speculated that whether we could load effective osteogenic agents into hASC‐derived exosomes to enhance bone formation.

miRNA is a type of endogenous small non‐coding RNA that often functioned through post‐transcriptional repression.^21^ Several miRNAs have been implicated in bone metabolism and osteogenic regulation.^22^, ^23^, ^24^ Our previous study has confirmed miR‐375 as a positive regulator in the osteogenic differentiation of MSCs, and overexpression of miR‐375 significantly enhanced the alkaline phosphatase (ALP) activity and calcium deposition in hASCs, suggesting that miR‐375‐mediated therapy might be a viable approach to repair bone defects.^25^ However, miRNAs tend to be easily degraded by RNase in vivo and have a short half‐life, which limits their application in bone tissue engineering.^26^ With the development of cell‐free transplantation strategy, we considered whether hASC‐derived exosomes could be applied as a carrier of osteogenic miRNA to achieve a combination of their functions and effects. In this study, we aimed to investigate whether exosomes derived from miR‐375‐overexpressing hASCs could enhance the therapeutic effects of bone regeneration and provide a basis for the application of exosomes as a gene delivery vehicle to transport therapeutic miRNAs for regenerative therapy.

## 2 MATERIALS AND METHODS

### 2.1 Cell culture

Primary hASCs and human bone marrow mesenchymal stem cells (hBMSCs) were obtained from ScienCell Company. Cells were cultured at 37°C in an incubator with 5% CO_2_ atmosphere and full relative humidity. To minimize the exogenous exosomes, hASCs were cultured in Dulbecco's Modified Eagle Medium (DMEM, Gibco) free of exosomes through ultracentrifugation at 100 000 *g* overnight with an Optima L‐90K Ultracentrifuge (Beckman Coulter, Inc). For the in vitro experiments, hBMSCs were cultured in proliferation medium (PM), which consisted of minimum essential medium α (α‐MEM, Gibco), 10% (v/v) foetal bovine serum (FBS, ScienCell) and 100 IU/mL antibiotics (Gibco). For osteogenic induction, hBMSCs were cultured in osteogenic medium (OM), which consisted of standard PM supplemented with 10 mmol/L β‐glycerophosphate, 0.2 mmol/L L‐ascorbic acid and 100 nmol/L dexamethasone. All other materials were purchased from Sigma‐Aldrich unless otherwise mentioned, and all experiments conducted with hBMSCs were extracted from three donors (Catalog#15901, #6881, #6890).

### 2.2 Lentiviral infection

Lentiviruses H1/GFP&Puro containing pre‐miR‐375 and the negative control (NC) were produced by GenePharma Company, and the sequences were provided as follows: pre‐miR‐375:5′‐CCCCGCGACGAGCCCCTCGCACAAACCGGACCTGAGCGTTTTGTTCGTTCGGCTCGCGTGAGGC‐3′; NC: 5′‐TTCTCCGAACGTGTCACGT‐3′. Lentiviral infection was performed according to our previous research.^25^

### 2.3 Isolation and purification of exosomes derived from hASCs

For exosome isolation, medium free of exosomes were replaced when hASCs arrived at a confluence of 80%, and the supernatants were collected 48 hours later. Exosomes were extracted from supernatants of hASCs by differential centrifugation and filtration steps.^17^, ^27^ Briefly, cell supernatants were centrifuged for 20 minutes at 2000 *g* and 40 minutes at 10 000 *g*, followed by filtering with a 0.22‐μm sterilized filter (Millipore). The supernatants were then ultracentrifuged for 70 minutes at 100 000 *g* and resuspended in phosphate‐buffered saline solution (PBS) for 70 minutes at 100 000 *g*. To remove any residual RNA, the pelleted exosomes were eluted in a mixture containing PBS and RNase I (Invitrogen). For the evaluation of exosomal concentration, exosomes were lysed in RIPA lysis buffer, and a Pierce bicinchoninic acid (BCA) Protein Assay Kit (Thermo Scientific) was used according to the manufacturer's instructions.

### 2.4 Identification of exosomes derived from hASCs

The morphology of exosomes was observed by transmission electron microscopy (TEM). hASC‐derived exosomes were fixed with 2% paraformaldehyde for 30 minutes and then dropped on carbon‐coated copper grids. After drying in air, the mixture was subjected to negative staining by using 1% uranyl acetate twice. Images were captured using an HT7700 TEM (Hitachi) at 120 kV.

The particle size and exosome concentration were determined by nanoparticle tracking analysis (NTA). Exosomes were measured using ZetaView system (Particle Metrix), and the results were analysed by NTA analytical software (zetaview, version 8.04.02) according to the manufacturer's instructions.

Specific markers—CD9, CD63, β‐tubulin and histone 1 were detected with western blotting to characterize hASC‐derived exosomes.

### 2.5 Exosome uptake assay

Exosomes were labelled with the red fluorescent cell linker PKH26 according to the manufacturer's instructions. Briefly, 20 μL exosomes isolated from miR‐375‐overexpressing hASCs (Exo [miR‐375]) at 25, 50 or 100 μg/mL were diluted in 1 mL diluent C and 4 μL PKH26 dye was diluted in 1 mL diluent C. The dilutions were then mixed gently for 4 minutes, and 2 mL of 0.5% bovine serum albumin (BSA) was added to bind excess dye. The labelled exosomes were washed in PBS at 100 000 *g* for 70 minutes. hBMSCs were then incubated with different concentrations of labelled exosomes for 4 or 24 hours. After incubation, the cells were washed twice with PBS and fixed in 4% paraformaldehyde for 10 minutes. Cellular nuclei were stained with 6‐diamidino‐2‐phenylindole (DAPI) solution at 1 μg/mL. The exosome uptake images were captured with an LSM 5 EXCITER confocal laser scanning microscope (Carl Zeiss).

### 2.6 Proliferation and osteogenic differentiation assays

Based on the dosage optimization, hBMSCs were treated with Exo (miR‐375) at 50 μg/mL, and exosomes isolated from hASCs expressing the control vector (Exo [NC]) were used as the control group. The culture media containing Exo (miR‐375) or Exo (NC) was replaced every 3 days. For cell proliferation assay, a cell‐counting kit‐8 (CCK‐8, Dojindo) was used to evaluate the cell number under the manufacturer's instructions, and the growth curve was formulated according to the absorbance values for 7 days.

For osteogenic differentiation assays, cells were cultured in PM or OM for 7 days and assayed for ALP staining and quantification. An NBT/BCIP staining kit (CoWin Biotech) was used after cell fixation to obtain ALP staining. An ALP assay kit (Nanjing Jiancheng Bioengineering Institute) was used to measure the ALP concentration, and total protein contents were determined in the same samples by using a Pierce BCA Protein Assay Kit (Thermo Scientific). ALP levels relative to the control group were calculated after normalization to the total protein contents. Cells cultured in PM or OM for 14 days were subjected to matrix mineralization as previously described.^27^ Cells were stained with 1% Alizarin red S (ARS, pH 4.2) at room temperature after fixing in 95% ethanol for 30 minutes. For quantification of mineralization, the stains were then dissolved in 100 mmol/L cetylpyridinium chloride for 30 minutes and determined at a 562 nm of absorbance value. The final mineralization levels in each group were calculated after normalizing to the total protein concentrations obtained from duplicate plates.

### 2.7 Quantitative reverse transcription PCR (qRT‐PCR)

Total RNAs of cells were extracted with TRIzol reagent (Invitrogen), and total RNAs of exosomes were isolated by using an miRNeasy Mini Kit (QIAGEN). The cDNA was reverse transcribed with a Reverse Transcription System (Takara), and qRT‐PCR was conducted using a 7500 Real‐Time PCR Detection System (Applied Biosystems) with a Power SYBR Green Master Mix (Roche) according to the following settings: 95°C for 10 minutes, 40 cycles of 95°C for 15 seconds and 60°C for 1 minutes. The internal standards for miR‐375 and mRNA were U6 and *GAPDH*, respectively. The primer sequences used are listed in Table S1, and the results were then analysed with the 2^−ΔΔ^ *^C^* ^t^ relative expression method.^28^

### 2.8 Reporter vector construction and dual‐luciferase reporter assay

We used the RNA22 software to predict the functional alignment of the target region of *insulin‐like growth factor binding protein 3* (*IGFBP3*). The 3′‐UTR of *IGFBP3* containing the predicted miR‐375 binding sites was synthesized and then cloned into a modified version of pcDNA3.1(+) containing a firefly luciferase reporter gene at a position downstream of the luciferase reporter gene to construct an *IGFBP3*‐wild‐type (WT) luciferase reporter plasmid. A Site‐Directed Mutagenesis Kit (SBS Genetech Co., Ltd) was then used to mutate the miR‐375 binding site in the 3ʹ‐UTR of *IGFBP3* and named as *IGFBP3*‐mutant‐type (MT) luciferase reporter plasmid. All constructs were confirmed by DNA sequencing. Luciferase reporter assays were performed using the methods described previously.^22^ Briefly, 293T were grown in a 48‐well plate till 70%‐80% confluence. 400 ng of plasmid expressing the IGFBP3‐MT or IGFBP3‐WT was transfected to the cells, along with 40 ng of the firefly luciferase reporter plasmid, and 4 ng of pRL‐TK, a plasmid‐expressing Renilla luciferase (Promega). Dual‐Luciferase Reporter Assay System was applied to measure luciferase activity 24 hours after transfection. All luciferase values were normalized to those of Renilla luciferase and expressed as fold change relative to basal activity.

### 2.9 Western blotting

Western blotting was conducted as previously described.^25^ Briefly, proteins from cells or exosomes were separated on an SDS‐PAGE gel and subsequently transferred to polyvinylidene difluoride membranes. Thereafter, the membranes were incubated with primary antibodies against CD9 (#ab92726, Abcam), CD63 (#ab134045, Abcam), β‐tubulin (#sc‐5274, Santa Cruz Biotechnology, Inc), histone 1 (#sc‐8030, Santa Cruz Biotechnology), IGFBP3 (#25864, Cell Signaling Technology) and GAPDH (#ab9485, Abcam) at 4°C overnight, and the secondary antibodies against rabbit (#7074, Cell Signaling Technology) and mouse (#7076, Cell Signaling Technology) were incubated for 1 hour at room temperature. An ECL kit (CoWin Biotech) was used to visualize the protein bands.

### 2.10 Construction and characterization of hydrogel loaded with exosomes

Hydrogel was obtained from Glycosan Biosystems, and it consisted of thiol‐modified hyaluronan, hydroxyapatite and thiol‐modified heparin, which can be crosslinked in situ.^29^ 20 μL Exo (miR‐375) or Exo (NC) at 50 μg/mL were mixed with 250 μL hydrogel following the manufacturer's instructions. An equal volume of hydrogel was used for the negative control. To observe the distribution of exosomes in the hydrogel, Exo (miR‐375) or Exo (NC) were labelled with PKH26, and the images were captured using the LSM 5 EXCITER confocal laser scanning microscope (Carl Zeiss). To further determine the release efficiency of exosomes in the hydrogel, the exosome‐loaded hydrogel was incubated in saline‐buffered solution at 37°C. The solution was collected every day to detect the remnant exosomal concentration with a Pierce BCA Protein Assay Kit (Thermo Scientific).

### 2.11 Animal experiments

A total of 36 male Sprague Dawley rats which weighed between 250 and 300 g were obtained from Vital River Laboratories and randomly divided into three groups (12 each). Animals were fed in a standard room with controlled temperature and humidity in a 12 hourly cycle of light and darkness. All animal experiments were approved by the Institutional Animal Care and Use Committee of the Peking University Health Science Center (Permit Numbers: LA 2014233) and performed according to the institutional animal guidelines. In situ skull defect experiments were conducted as described previously.^17^ With copious saline irrigation, calvarial defects with 5 mm diameter were constructed using a trephine bur (Hager Meisinger GmbH) under low‐speed drilling. All the defects on the left side were left as the blank group without any treatment, and defects on the right side were implanted with hydrogel, hydrogel loaded with Exo (NC) at 50 μg/mL or hydrogel loaded with Exo (miR‐375) at 50 μg/mL. To check the existence of exosomes in the defect sites, immunohistochemical (IHC) staining against CD63 (#ab134045, Abcam) which merely reactivated to human was conducted at 3 days, 2, 4 and 8 weeks after implantation.

### 2.12 Analysis of bone regeneration in vivo

Eight weeks after surgery, the whole calvarium including the implants was surgically removed and fixed in 4% paraformaldehyde. To assess the ability of bone formation, the specimens were scanned with high‐resolution Inveon micro‐computed tomography (μCT, Siemens) following the experimental settings: 80 kV x‐ray voltage, 500 μA node current and 1500 ms exposure time for each of the 360 rotational steps. Then, three‐dimensional (3D) images were reconstructed with multimodal 3D visualization software, and bone volume/total volume (BV/TV) and bone mineral density (BMD) were calculated using the Inveon Research Workplace software. Thereafter, the samples were decalcified in 10% EDTA (pH 7.4) for 14 days and embedded in paraffin after dehydration. 5 μm sections were cut and used for haematoxylin and eosin (HE) and Masson staining. IHC staining was also performed with primary antibodies against osteocalcin (#ab13420, Abcam), BMP2 (#18933‐1‐AP, proteintech) and IGFBP3 (#10189‐2‐AP, proteintech). Tissue slices were visualized under a light microscope (Olympus).

### 2.13 Statistical analysis

Results were analysed using the spss 20.0 software (IBM). Data from three independent experiments were presented as mean ± standard deviation (SD). Comparisons between two groups were analysed by independent two‐tailed Student's *t* test. Comparisons between more than two groups were analysed by one‐way ANOVA followed by Turkey's test. A two‐tailed *P*‐value of <.05 was considered statistically significant.

## 3 RESULTS

### 3.1 Characterization of exosomes derived from modified hASCs

**Figure 1:**
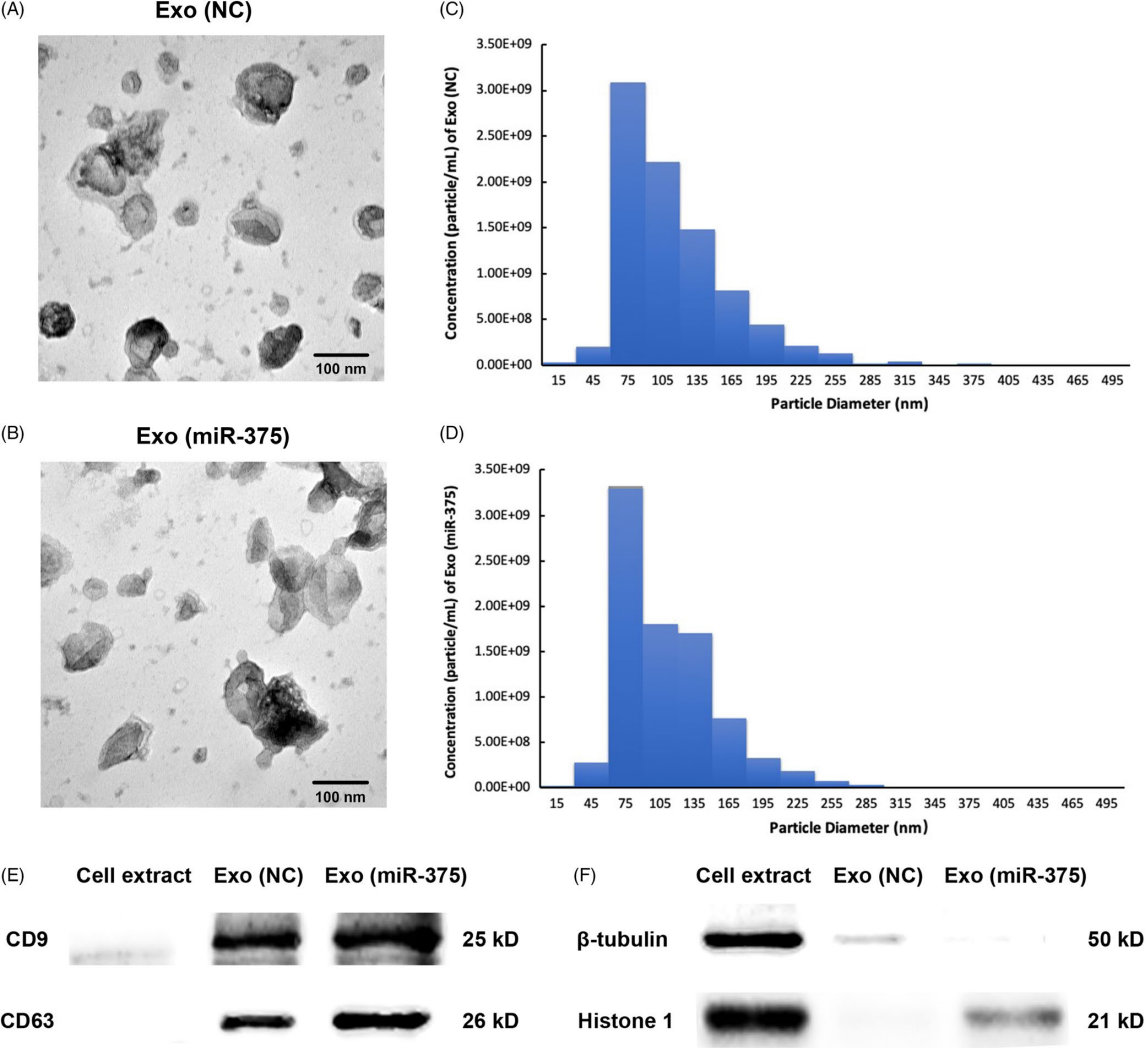
Characterization of exosomes from modified hASCs. A and B, Morphology of Exo (NC) and Exo (miR‐375) observed by TEM. Scale bars = 100 nm. C and D, Particle size distribution and concentration of Exo (NC) and Exo (miR‐375) measured by Nanosight analysis. E and F, Western blotting analysis of the exosomal surface markers, cytosolic marker and nuclear marker from Exo (NC) and Exo (miR‐375) compared with cell extraction. TEM, transmission electron microscopy

To specifically modify the contents of exosomes, we first generated hASCs stably overexpressing miR‐375, and the transduction efficiency was confirmed with micrographs and qRT‐PCR (Figure S1). TEM analysis showed that Exo (NC) and Exo (miR‐375) exhibited spherical morphology (Figure 1A and B). NTA analysis indicated that the size of Exo (NC) and Exo (miR‐375) was mostly distributed around 75 nm (Figure 1C and D), generally corresponding to the exosome parameters in other reports.^17^, ^27^ Furthermore, western blotting confirmed the expression of CD9 and CD63 which are commonly recognized as exosomal markers (Figure 1E), with barely detectable expression of either β‐tubulin (cytosolic marker) or histone 1 (nuclear marker; Figure 1F).

### 3.2 Optimization of Exo (miR‐375) with hBMSCs

**Figure 2:**
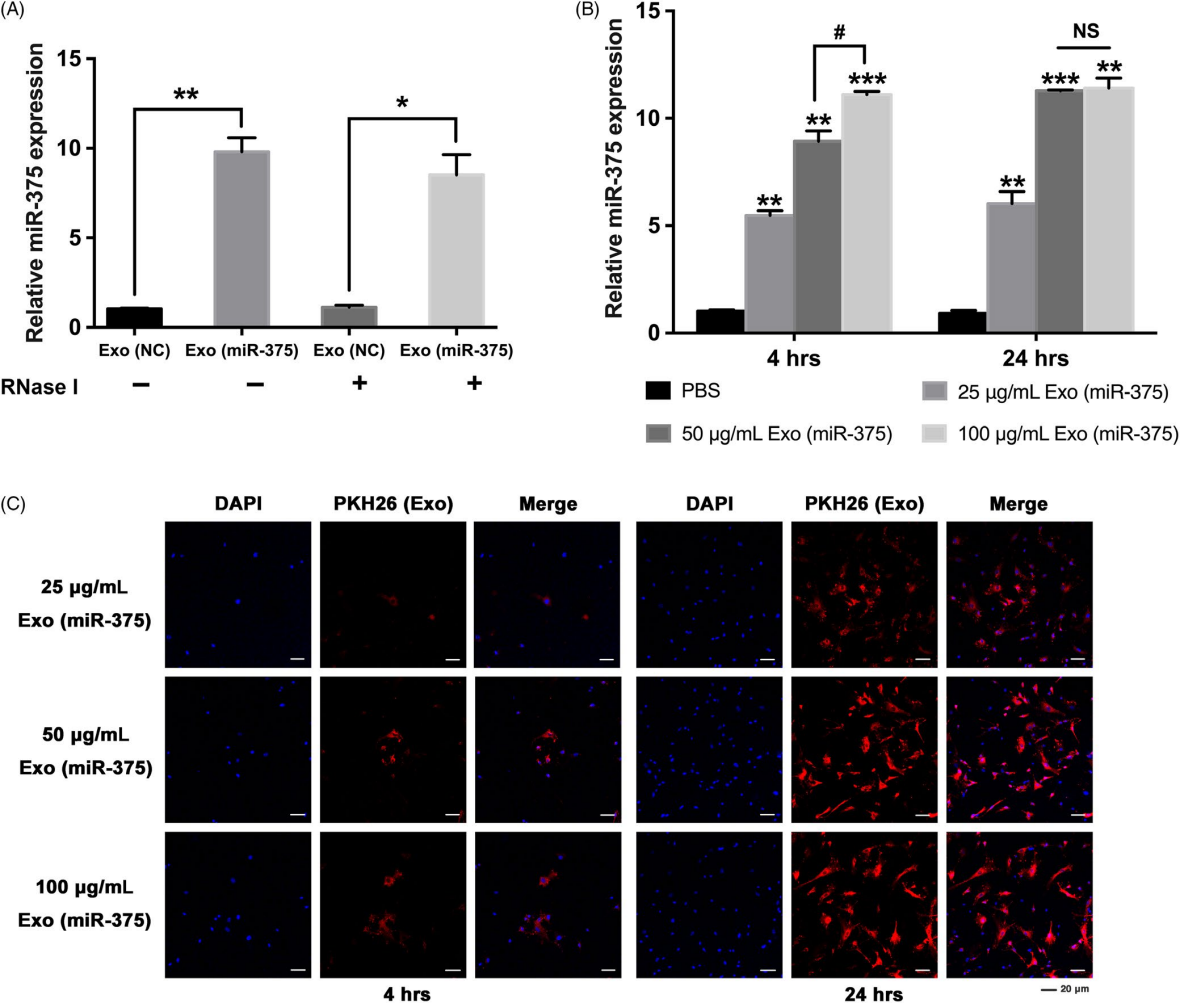
Optimization of Exo (miR‐375) with hBMSCs. A, Relative expression of miR‐375 in Exo (miR‐375) compared with Exo (NC) before and after RNase treatment with qRT‐PCR. U6 was used for normalization. B and C, Exo (miR‐375) was delivered into hBMSCs at different concentrations and incubated for 4 or 24 h. B, Relative expression of miR‐375 in hBMSCs determined by qRT‐PCR. U6 was used for normalization. C, Cellular internalization of Exo (miR‐375) by hBMSCs. The nucleus of hBMSCs was stained with DAPI (blue), and Exo (miR‐375) was labelled with PKH26 (red). Scale bars = 20 μm. Data are represented as mean ± SD; n = 3; *P < .05 compared with the control group; **P < .01 compared with the control group; ***P < .001 compared with the control group; ^#^ *P* < .05; NS: not significant. hBMSCs, human bone marrow mesenchymal stem cells; DAPI, 6‐diamidino‐2‐phenylindole

To confirm the contents of modified exosomes, qRT‐PCR analysis revealed that the expression of miR‐375 increased significantly in Exo (miR‐375) compared with Exo (NC) (Figure 2A). RNase was administrated in the final isolation procedure to exclude the pollution of exogenous RNA, and qRT‐PCR analysis indicated that the expression of miR‐375 also upregulated in Exo (miR‐375) after RNase treatment (Figure 2A). Exosomal concentration was then evaluated according to the protein level, and 25 μg/mL exosomes could be obtained from almost 100 mL hASC supernatants. qRT‐PCR analysis showed that Exo (miR‐375) treatment led to a remarkable increase in miR‐375 in hBMSCs for 4 hours, and the effect was dependent on dosage, while no significant difference was detected between the 50 μg/mL and 100 μg/mL groups when the incubation time prolonged to 24 hours (Figure 2B). Similarly, fluorescence microscopy revealed that PKH26‐labelled exosomes (red dots) were gradually internalized by hBMSCs as the incubation time prolonged, while there was not any noteworthy difference in the red dot numbers between the 50 μg/mL and 100 μg/mL groups (Figure 2C). Consequently, we selected Exo (miR‐375) at a concentration of 50 μg/mL for the subsequent assays.

### 3.3 Exo (miR‐375) promoted osteogenic differentiation of hBMSCs in vitro

**Figure 3:**
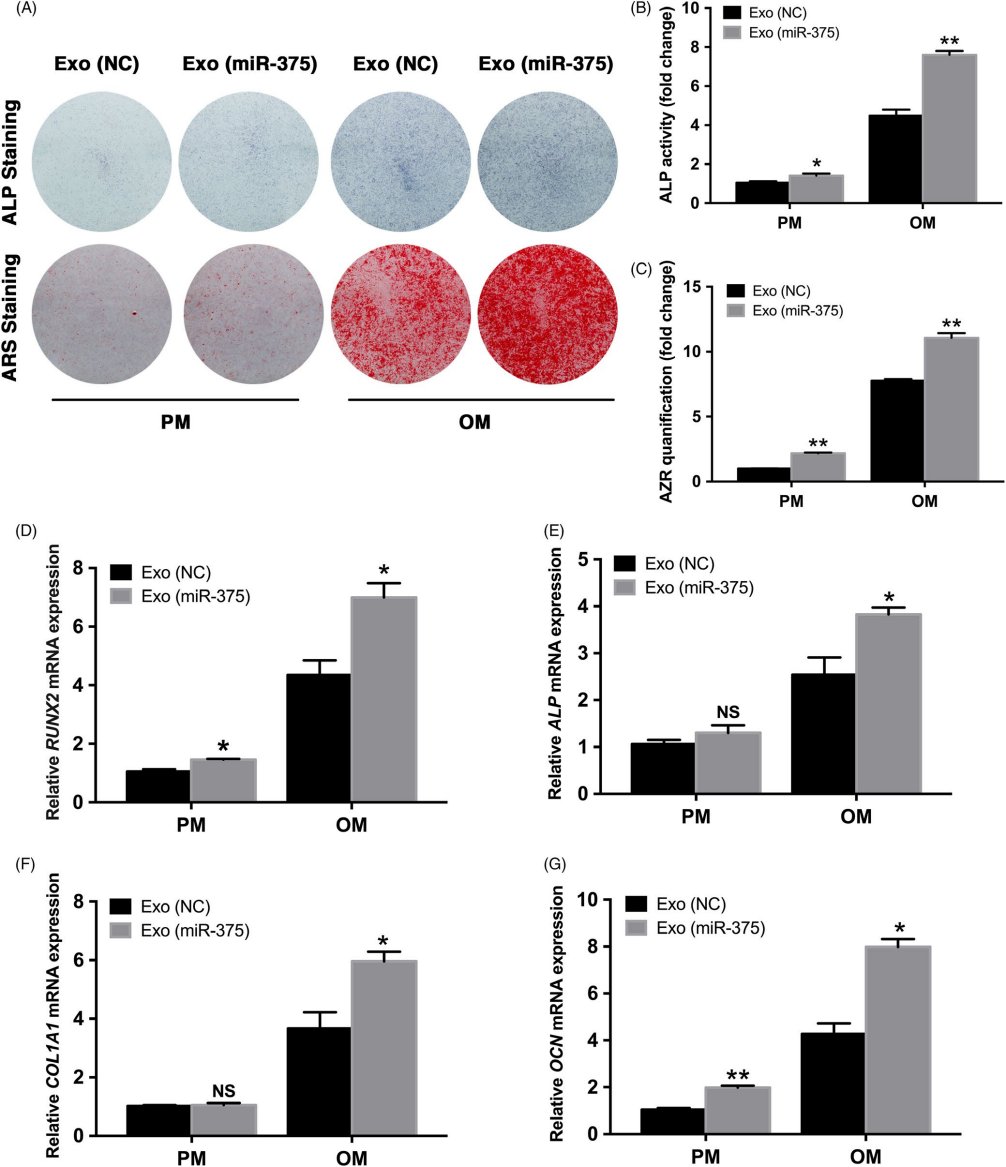
Exo (miR‐375) promoted osteogenic differentiation of hBMSCs in vitro. Exo (miR‐375) at 50 μg/mL was delivered into hBMSCs, and Exo (NC) at the same concentration was used as control. A, ALP staining on day 7 and ARS staining on day 14 in PM and OM. B and C, ALP activity on day 7 and ARS mineralization assay on day 14 in PM and OM. D and E, Relative mRNA expression of *RUNX2* and *ALP* on day 7 in PM and OM measured by qRT‐PCR *GAPDH* was used for normalization. F and G, Relative mRNA expression of *COL1A1* and *OCN* on day 14 in PM and OM measured by qRT‐PCR *GAPDH* was used for normalization. Data are represented as mean ± SD; n = 3; *P < .05; **P < .01; NS: not significant. ALP, alkaline phosphatase; ARS, alizarin red S; PM, proliferative medium; OM, osteogenic medium; RUNX2, runt related transcription factor 2; COL1A1, collagen type I alpha 1 chain; OCN, osteocalcin; GAPDH, glyceraldehyde‐3‐phosphate dehydrogenase

We first determined the proliferative ability of hBMSCs with Exo (miR‐375) at 50 μg/mL, and the results indicated that no significant difference was detected between Exo (miR‐375) and Exo (NC) groups (Figure S2). To further validate the role of Exo (miR‐375) in osteogenic differentiation, we treated hBMSCs under PM or OM with Exo (miR‐375), and Exo (NC) was delivered as the control group. After osteogenic stimulation for 7 days, ALP staining and activity in hBMSCs treated with Exo (miR‐375) was significantly enhanced compared with that in hBMSCs treated with Exo (NC) (Figure 3A and B). Furthermore, ARS staining and quantification on day 14 indicated that the extracellular matrix mineralization was also markedly elevated by treatment with Exo (miR‐375) (Figure 3A and C). Consistently, stimulation with Exo (miR‐375) upregulated the mRNA expression of osteogenesis‐related genes including *RUNX2*, *ALP*, *COL1A1* and *OCN* in hBMSCs with osteogenic induction (Figure 3D‐G).

### 3.4 Overexpression of miR‐375 inhibited *IGFBP3* by targeting its 3′‐UTR

**Figure 4:**
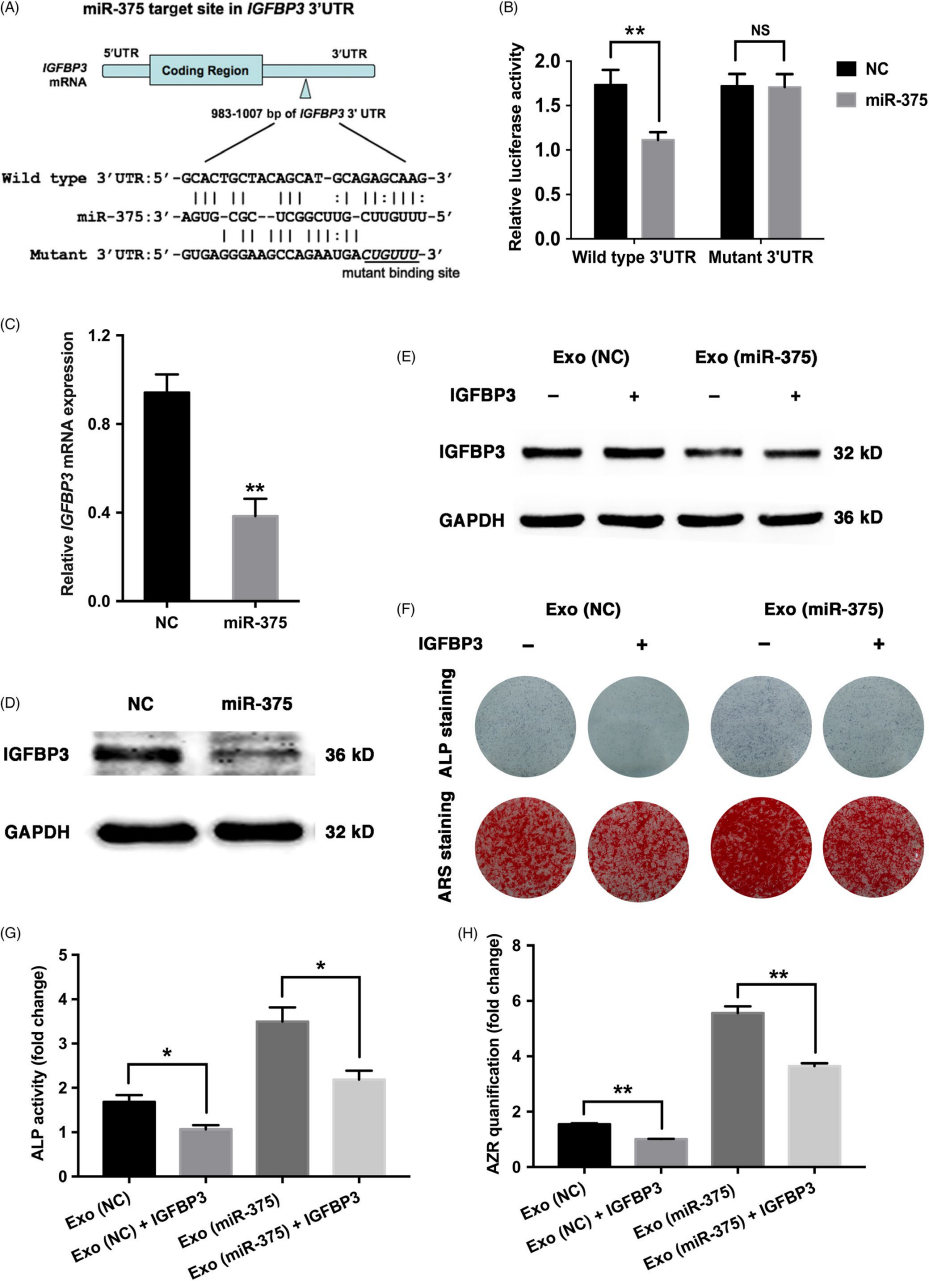
miR‐375 inhibited *IGFBP3* by targeting its 3′‐UTR. A‐D, hBMSCs were transfected with lentivirus overexpressing miR‐375, and NC was used as the control vector. A, Predicted binding sites of miR‐375 in the 3′‐UTR of *IGFBP3*‐WT mRNA (mutated bases in the 3′‐UTR of *IGFBP3*‐MT mRNA are underlined). B, Luciferase activity of cells with miR‐375 overexpression in the *IGFBP3*‐WT and *IGFBP3*‐MT groups. C, Relative mRNA expression of *IGFBP3* in the miR‐375 and NC groups determined by qRT‐PCR *GAPDH* was used for normalization. D, Western blotting of IGFBP3 in the miR‐375 and NC groups. GAPDH was used as the internal control. E and H, The recombinant protein IGFBP3 was delivered into hBMSCs with Exo (NC) or Exo (miR‐375). E, Western blotting of IGFBP3 expression. GAPDH was used as the internal control. F, ALP staining on day 7 and ARS staining on day 14 after osteogenic induction. (G‐H) ALP activity on day 7 and ARS mineralization assay on day 14 after osteogenic induction. Data are represented as mean ± SD; n = 3; *P < .05; **P < .01; NS: not significant. IGFBP3, insulin‐like growth factor binding protein 3

According to our previous transcriptome microarray, 67 genes were downregulated with miR‐375 overexpression (Table S2), and among these downregulated genes, we noticed a marked decrease in *IGFBP3*. As a critical regulator of cell differentiation, IGFBP3 has been investigated to play a role in osteogenic differentiation, and the level of IGFBP3 has correlated with bone‐related diseases.^30^, ^31^, ^32^ RNA22 prediction indicated the putative binding sites of miR‐375 in the 3′‐UTR of *IGFBP3* (Figure 4A). Luciferase activity analysis revealed that miR‐375 repressed luciferase expression of vectors containing the 3′‐UTR of wild‐type *IGFBP3*, but did not significantly affect mutant‐type *IGFBP3* (Figure 4B). Further analysis demonstrated that IGFBP3 expression was significantly inhibited in miR‐375‐overexpressing hASCs (Figure 4C and D). Considering that IGFBP3 belongs to the IGFBP family, which consists of 6 homogenous members, we then determined the expression of other members, and no obvious difference was detected in the relative mRNA levels of other members of the IGFBP family (including *IGFBP1*, *IGFBP2*, *IGFBP4*, *IGFBP5* and *IGFBP6*) between the miR‐375‐overexpressing hASCs and the control cells (Figure S3).

### 3.5 IGFBP3 reduced the osteogenic effects triggered by Exo (miR‐375)

Since IGFBP3 was directly regulated by miR‐375, we investigated its role in the osteogenic differentiation of hBMSCs. Two different sequences of small interfering RNA (siRNA) targeting *IGFBP3* were designed, and the knockdown efficiency was validated by western blotting analysis (Figure S4A). ALP staining and quantification showed that knockdown of *IGFBP3* accelerated the osteogenic differentiation of hBMSCs 7 days after osteogenic induction (Figure S4B and C). Moreover, extracellular matrix mineralization, determined by ARS staining and quantification, was increased in *IGFBP3* deficient cells 14 days after osteogenic induction (Figure S4B and D).

To elucidate the functional connection between miR‐375 and IGFBP3 during the osteogenic differentiation, we then administrated recombinant IGFBP3 protein in Exo (miR‐375)‐treated cells. Western blotting analysis showed that recombinant IGFBP3 protein was successfully delivered into hBMSCs (Figure 4E). As ALP staining and quantification indicated, the increase in osteogenic differentiation induced by Exo (miR‐375) treatment was effectively reversed by IGFBP3 (Figure 4F and G). Similar results were obtained when the extracellular matrix mineralization effect was assessed by ARS staining and quantification (Figure 4F and H).

### 3.6 Characterization of Exo (miR‐375) embedded with hydrogel

**Figure 5:**
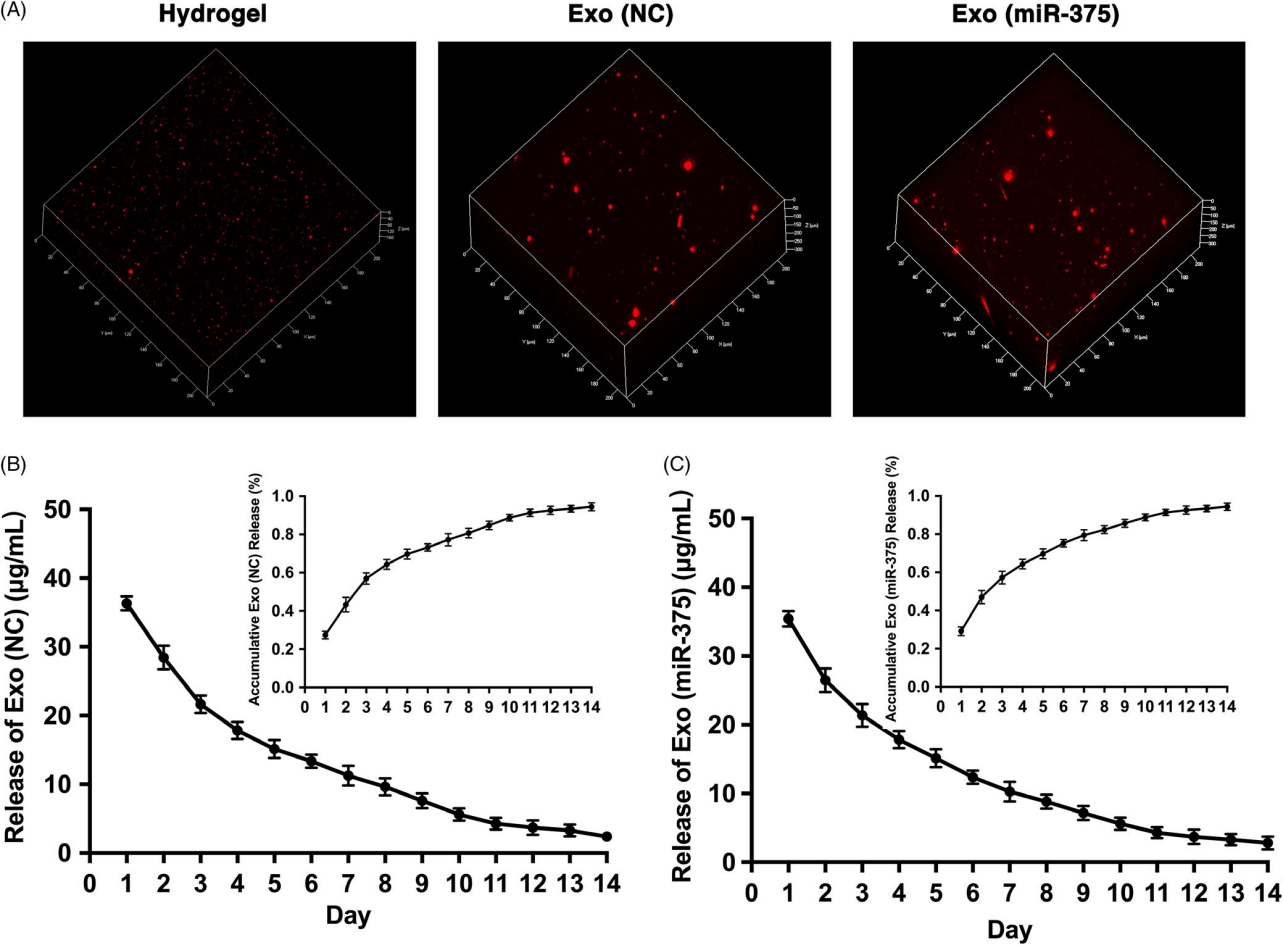
Characterization of Exo (miR‐375) embedded with hydrogel. A, Distribution of PKH26‐labelled exosomes in the hydrogel, with PKH26‐stained hydrogel as a negative control (Left). B and C, In vitro exosome release kinetics of Exo (NC) and Exo (miR‐375) in saline from exosome‐embedded hydrogel

To explore the effects of Exo (miR‐375) in vivo, we embedded Exo (miR‐375) or Exo (NC) with commercial hydrogel to construct tissue‐engineered bone. The exosomes were then labelled with PKH26, and PKH26‐stained hydrogel was used as a negative control. As shown in the fluorescence microscope image, Exo (miR‐375) and Exo (NC) were homogeneously distributed in the hydrogel, whereas there were few red dots on the PKH26‐stained hydrogel, suggesting the successful immobilization of exosomes (Figure 5A). Moreover, the release curve of the incorporated exosomes showed a slow and controlled release during the 14‐day monitoring span (Figure 5B and C).

### 3.7 Exo (miR‐375) enhanced bone formation in calvarial defects

**Figure 6:**
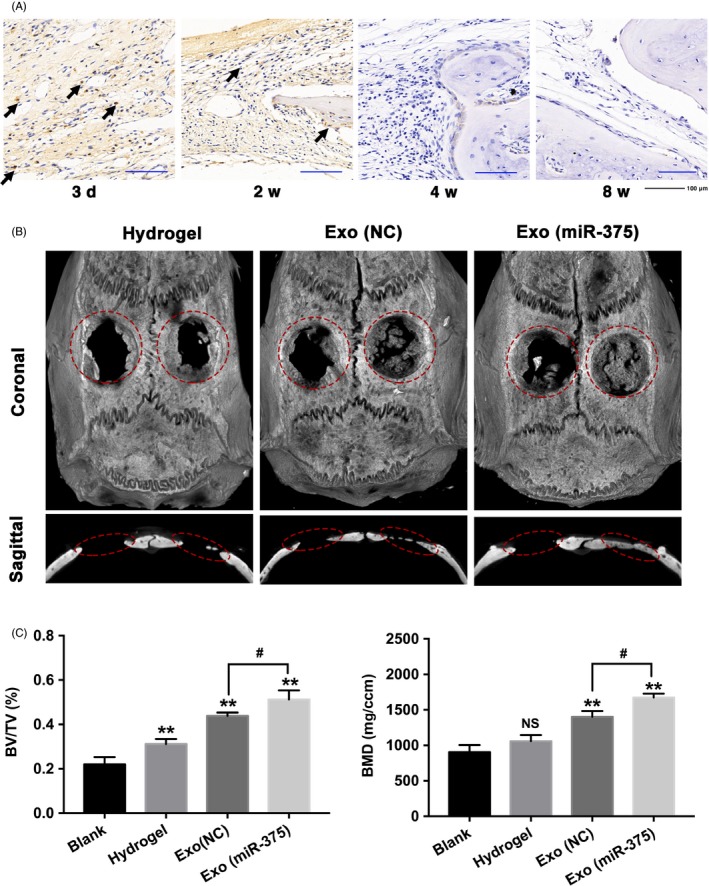
Exo (miR‐375) enhanced bone formation in a rat model of calvarial defect. A, IHC staining against CD63 at 3 d, 2, 4 and 8 wk after operation in Exo (miR‐375) group, and dark‐brown granules indicating positive staining are marked by black arrows. Scale bars = 100 μm. B and C, Defects on the left side were left as the blank group without any treatment, and defects on the right side were treated with hydrogel, hydrogel loaded with Exo (NC), and hydrogel loaded with Exo (miR‐375). B, The 3D reconstruction images in each group 8 wk after operation. C, Analysis of BV/TV and BMD in each group. Data are represented as mean ± SD; n = 12; **P < .01 compared with the blank group; ^#^ *P* < .05; NS: not significant. BV/TV, bone volume/total bone volume; BMD, bone mineral density

To further evaluate the biological role of Exo (miR‐375) in bone formation, we introduced a rat model of calvarial defects. Hydrogel loaded with Exo (NC) or Exo (miR‐375) at 50 μg/mL was implanted on the right side of the calvarial defect, and the bare hydrogel was used as the control group. All the left defects were left as the blank group without any treatment. IHC staining against CD63 indicated that the exosomes were distributed in the defect sites 3 days after implantation and existed at least for 2 weeks (Figure 6A). 3D reconstruction revealed that the new bone formation in Exo (miR‐375) group was greater than that in Exo (NC) group from both the coronal and sagittal views (Figure 6B). Quantification of μCT images showed that the proportion of BV/TV and BMD were significantly increased with Exo (miR‐375) treatment (Figure 6C).

**Figure 7:**
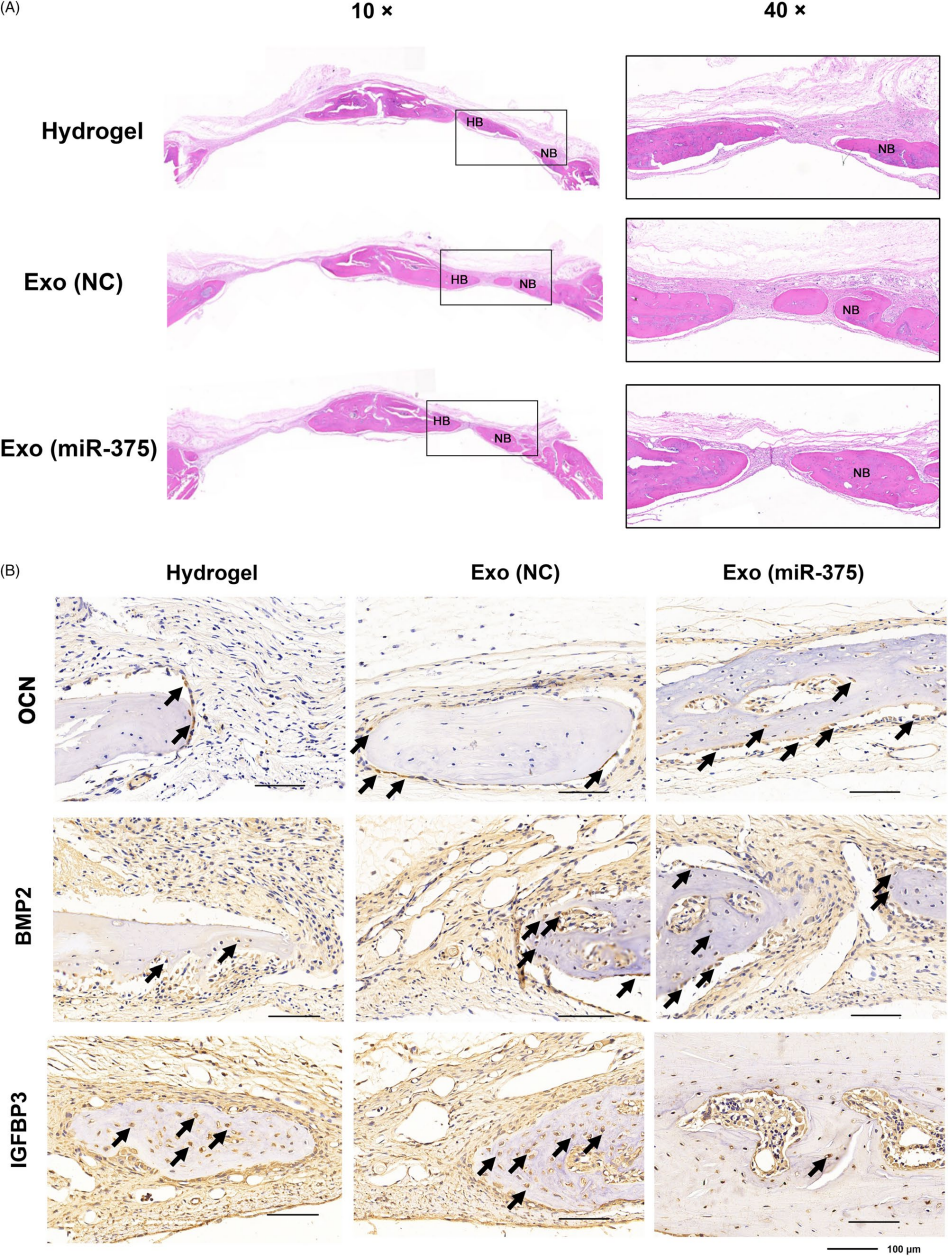
Histological evaluation of the newly formed bone 8 wk after operation. A, HE staining in each group with the magnification at 10× and 40×. HB, host bone; NB, new bone. B, IHC staining against OCN, BMP2 and IGFBP3 in each group, and dark‐brown granules indicating positive staining are marked by black arrows. Scale bars = 100 μm

As for the histological examination, HE staining revealed that more newly formed bone tissues were present along the defect margin in Exo (miR‐375) group than that in Exo (NC) group (Figure 7A), and the osteoid accumulated in Exo (miR‐375) group appeared more mature as Masson's trichrome staining referred (Figure S5). Moreover, IHC staining against OCN and BMP2 indicated that the range and intensity of the stained granules around the nucleus or in the cytoplasm of osteoblasts were higher in Exo (miR‐375) group (Figure 7B). To ascertain the mechanism of miR‐375 in vitro, we further determined the expression of IGFBP3 in situ, and the result showed that less IGFBP3 was observed in osteoblasts with Exo (miR‐375) administration (Figure 7B).

## 4 DISCUSSION

Gene therapy in regenerative medicine relies on nano‐sized vectors for the efficient delivery of specific cargo to specified target sites.^33^ Viral and non‐viral nanocarriers have been engineered to realize effective and site‐specific delivery.^3^ Nevertheless, the drawbacks of viral delivery, such as non‐specific cytotoxicity, poor biocompatibility and inefficient delivery, remain a major challenge. Exosomes, as naturally produced biological carriers, have therefore become a preferred option for nano‐scale delivery.^34^

Exosomes often display characteristics similar to those of their parent cells when encapsulated with various molecular constituents from the originating cells. Selection of producer cell type for therapeutic application is therefore of great importance. hASCs, a type of MSCs, are considered an ideal cell source for exosome production, owing to their rapid proliferation, low immunogenicity and high stability.^15^, ^16^, ^35^ Exosomes derived from hASCs accelerated cutaneous wound healing by optimizing the properties of fibroblasts.^36^ Moreover, exosomes released from ASCs showed therapeutic potential in the treatment of ischaemic diseases by promoting angiogenesis.^37^ In contrast to cell‐based regeneration, exosome‐based therapy can be safer in application. With lower concentration of membrane‐bound proteins, such as major histocompatibility complex (MHC) molecules, exosomes are less immunogenic than their parent cells.^38^ Moreover, exosomes can encapsulate and prevent the rapid degradation of small soluble molecules, such as cytokines, transcription factors and RNAs.^18^, ^39^

Effective loading of bioactive agents into exosomes remains a critical problem in exosome‐mediated therapeutic delivery. There are currently two main strategies for cargo loading—electroporation and genetic modification.^39^, ^40^ Exosomes and siRNA cargo tend to aggregate during electroporation, which considerably reduces the loading efficiency.^41^ It remains ambiguous whether other RNA molecules, such as miRNAs and mRNAs, could be effectively loaded by electroporation. In this study, we loaded miR‐375 into hASC‐derived exosomes by genetic modification of hASCs. This loading strategy has been previously reported to incorporate natural miRNAs, small hairpin RNAs (shRNAs) and mRNAs into exosomes.^42^, ^43^ Exosomes derived from miR‐181‐5p‐modified ASCs exhibited an accumulation of miR‐181‐5p and prevented liver fibrosis in a mouse model.^19^ By expressing high levels of the suicide gene mRNA and protein‐cytosine deaminase (CD) fused to uracil phosphoribosyltransferase (UPRT) in donor cells, microvesicles were successfully engineered with CD‐UPRT mRNA/protein and contributed to the regression of schwannomas.^18^ Here, we demonstrated that miR‐375 could be enriched in hASC‐derived exosomes by overexpressing the miRNA cargo in the parent cells and remained stable with RNase treatment.

Once released from the parent cells, exosomes can be transferred to recipient cells, where cargo delivery occurs.^44^ The binding of exosomes to recipient cells is mainly mediated by ligand‐receptor recognition; however, the underlying mechanism remains unclear. To optimize targeting activity, specific moieties may be engineered on the vesicle surface of exosomes. For example, exosomes modified with epidermal growth factor (EGF) on the surface could efficiently transfer genes to cancer tissues expressing epidermal growth factor receptor (EGFR).^45^ In this study, we observed that exosomes enriched with miR‐375 were successfully internalized by the recipient cells, as indicated by the increased expression of miR‐375 in hBMSCs. A miRNA could bind the 3′‐UTR of several genes. Our previous study reported that miR‐375 promoted osteogenic differentiation by targeting the 3′‐UTR of *DEPTOR*, and in this study, we confirmed that *IGFBP3* is another target gene. IGF (insulin‐like growth factor) signalling, a crucial pathway mediating skeletal growth, is regulated by 6 binding proteins, IGFBP1‐6, which can activate or repress IGF action locally.^46^ In this study, we demonstrated that IGFBP3 acts as a negative regulator of osteogenic differentiation and that exosomes enriched with miR‐375 could deliver miRNA cargo to hBMSCs, and thereby inhibit the expression of *IGFBP3* to exert osteogenic effects.

To achieve optimal in vivo application, the pharmacokinetics and distribution of exosomes should be taken into consideration. After intravenous injection, large quantities of labelled exosomes were distributed in the spleen, liver, lung and kidney after 30 minutes, and the half‐life of injected exosomes was almost 3 hours in blood.^47^ Because of its rich vascularity, the spleen tends to accumulate large quantities of exosomes.^48^ The intravenous application of exosomes shows considerable promise in the treatment of tumours, owing to the enhanced permeability and leaky vasculature of tumour cells.^49^ In contrast to systemic administration, locally administered exosomes could show high concentrations at target sites, especially in sites with poor vasculature. We constructed genetically modified exosomes with hydrogel for local administration in calvarial defects. Hydrogel has been widely used as scaffolds for tissue regeneration because of their unique features, such as high biocompatibility, slow release of engineered factors and modulated 3D networks.^50^ The engineered hydrogel in our study exhibited a slow and controlled release of exosomes, and further in vivo analysis demonstrated that Exo (miR‐375) embedded with hydrogel enhanced the bone regenerative capacity in a rat model of calvarial defect. However, some problems need to be addressed. Firstly, more effective and convenient loading strategies should be developed, since loading specific cargo via genetic modification of the parent cells involves multiple biogenesis procedures, such as RNAs sorting, the mechanism of which remains ambiguous. Further, robust immune profiling following exosome administration should be performed to determine the immune response of the recipient, which can provide guidance for clinical application.

## CONFLICT OF INTEREST

The authors declare no competing financial interest.
