# Supplementary material for: Exosomes derived from miR‐375‐overexpressing human adipose mesenchymal stem cells promote bone regeneration
Source: Cell Prolif. 2019 Aug 5;52(5):e12669. doi: 10.1111/cpr.12669 (PMC6797519; doi:10.1111/cpr.12669)
Supplement: Supplementary file 1 [file CPR-52-e12669-s001.docx]

**Table S1. Sequences of RNA and DNA Oligonucleotides**

| **Name** | **Sense Strand/Sense Primer (5'-3')** | | | **Antisense Strand/Antisense Primer (5'-3')** | | | |  |  |  |
| --- | --- | --- | --- | --- | --- | --- | --- | --- | --- | --- |
| **siRNA** | | | | | | | | | | |
| IGFBP3-1 | | GUCAUCAUCUCAAGACCUATT | | | UAGGUCUUGAGAUGAUGACTT | |  |  |  |  |
| IGFBP3-2 | | GUCUGUCAGUUUGUCGUCUTT | | | AGACGACAAACUGACAGACTT | |  |  |  |  |
| *NC* | | UUCUCCGAACGUGUCACGUTT | | | ACGUGACACGUUCGGAGAATT | |  |  |  |  |
| **Primers for qRT-PCR** | | | | | | | | | | |
| miR-375 RT primer GCTGTCAACGATACGCTACCTAACGGCATGACAGTGTCAGCCTA | | | | | | | | | | |
| miR-375 | | | GTGCAGGGTCCGAGGT | | | AGCCGTTTGTTCGTTCGGCT | | | |  |
| U6 | | | CTCGCTTCGGCAGCACA | | | AACGCTTCACGAATTTGCGT | | | |  |
| *RUNX2* | | | CCGCCTCAGTGATTTAGGGC | | | GGGTCTGTAATCTGACTCTGTCC | | | |  |
| *ALP* | | | ATGGGATGGGTGTCTCCACA | | | CCACGAAGGGGAACTTGTC | | | |  |
| *COL1A1* | | | ACAGGGCTCTAATGATGTTGA | | | AGGCGTGATGGCTTATTTGT | | | |  |
| *OCN* | | | CACTCCTCGCCCTATTGGC | | | CCCTCCTGCTTGGACACAAAG | | | |  |
| *IGFBP3* | | | AGAGCACAGATACCCAGAACT | | | GGTGATTCAGTGTGTCTTCCATT | | | |  |
| *IGFBP1*  *IGFBP2*  *IGFBP4*  *IGFBP5*  *IGFBP6* | | | TTGGGACGCCATCAGTACCTA  GACAATGGCGATGACCACTCA  GGTGACCACCCCAACAACAG  ACCTGAGATGAGACAGGAGTC  AGGAGTGCGGGGTCTACAC | | | TTGGCTAAACTCTCTACGACTCT  CAGCTCCTTCATACCCGACTT  GAATTTTGGCGAAGTGCTTCTG  GTAGAATCCTTTGCGGTCACAA  CTCTGCGGTTCACATCCTGT | | | |  |
| *GAPDH* | | | GAAGGTGAAGGTCGGAGTC | | | GAAGATGGTGATGGGATTTC | | |  |  |

**Table S2. The expression of downregulated genes with miR-375 overexpression by microarray analysis (*P* < 0.001).**

| Gene | Ratio | Gene | Ratio | Gene | Ratio |
| --- | --- | --- | --- | --- | --- |
| *DEPTOR* | 0.3794 | *MGARP* | 0.404 | *MEST* | 0.4194 |
| *FRZB* | 0.4449 | *INMT* | 0.4455 | *SERPINB2* | 0.4468 |
| *HSPB7* | 0.4697 | *FAM43A* | 0.4709 | *EFHD1* | 0.473 |
| *KRTAP1-5* | 0.4809 | *ELN* | 0.4836 | *PDE1A* | 0.5072 |
| *RGCC* | 0.508 | *SCUBE3* | 0.517 | *MGP* | 0.5265 |
| *CTSC* | 0.5398 | *CDKN1C* | 0.5431 | ***IGFBP3*** | **0.5474** |
| *EGR1* | 0.5536 | *FHL1* | 0.5583 | *STC1* | 0.5653 |
| *NREP* | 0.5702 | *SYNPO2* | 0.5736 | *ROR1* | 0.578 |
| *COMP* | 0.5801 | *TPD52L1* | 0.5831 | *NGF* | 0.5848 |
| *TNXA* | 0.586 | *CLIC3* | 0.5865 | *DAPK1* | 0.5892 |
| *INHBA* | 0.5907 | *GATA6* | 0.598 | *TLR4* | 0.5998 |
| *TMEM200A* | 0.603 | *KRT7* | 0.6086 | *CLEC3B* | 0.6127 |
| *SEL1L3* | 0.615 | *FAM107A* | 0.616 | *MN1* | 0.618 |
| *MFAP5* | 0.6192 | *RHOB* | 0.6204 | *NABP1* | 0.6211 |
| *PRSS23* | 0.6217 | *MEGF6* | 0.6225 | *C1orf198* | 0.6251 |
| *PPP1R14A* | 0.6277 | *GREM2* | 0.628 | *LIMCH1* | 0.6283 |
| *FHL2* | 0.6283 | *CPZ* | 0.6287 | *RDH10* | 0.6318 |
| *CKB* | 0.635 | *DBNDD2* | 0.6359 | *SERPINE2* | 0.6359 |
| *PLAGL1* | 0.6366 | *BAMBI* | 0.6447 | *SUGCT* | 0.6493 |
| *STC2* | 0.6505 | *PGM1* | 0.6524 | *SVEP1* | 0.6539 |
| *PPAP2B* | 0.6558 | *ALDH1A3* | 0.6588 | *PHACTR2* | 0.6601 |
| *NATD1* | 0.6606 | *SLC40A1* | 0.6622 | *NTN4* | 0.6628 |
| *SORBS2* | 0.6636 |  |  |  |  |


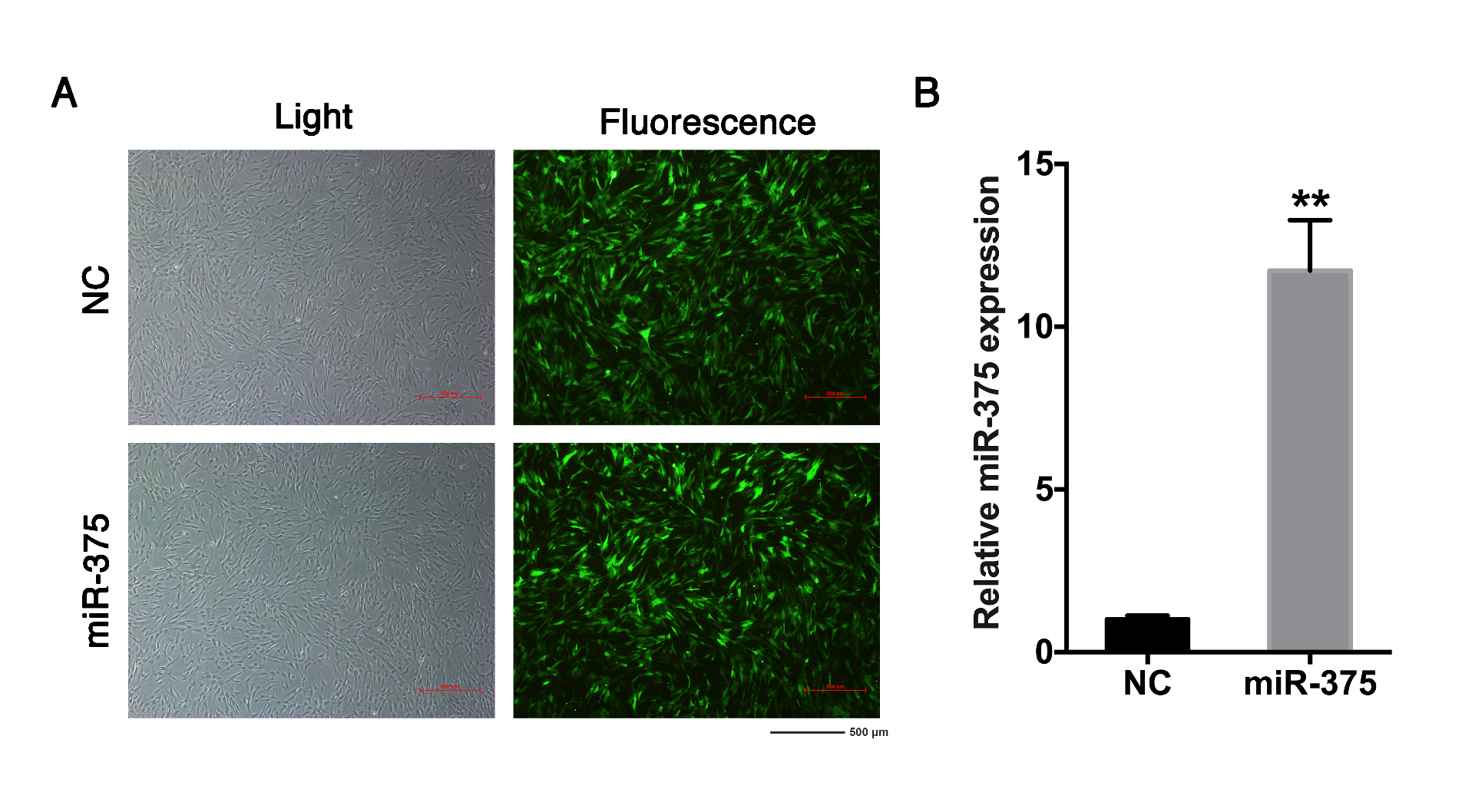


**Figure S1.** Transduction efficiency of lentivirus containing miR-375 or control vector. (A) Micrographs of GFP-positive hASCs under ordinary and fluorescent light. scale bars = 500 μm. (B) Relative miR-375 expression in miR-375 and NC groups as determined by qRT-PCR. U6 was used for normalization. Data are represented as mean ± SD; n = 3; ***P* < 0.01. GFP, green fluorescent protein; hASCs, human adipose mesenchymal stem cells.


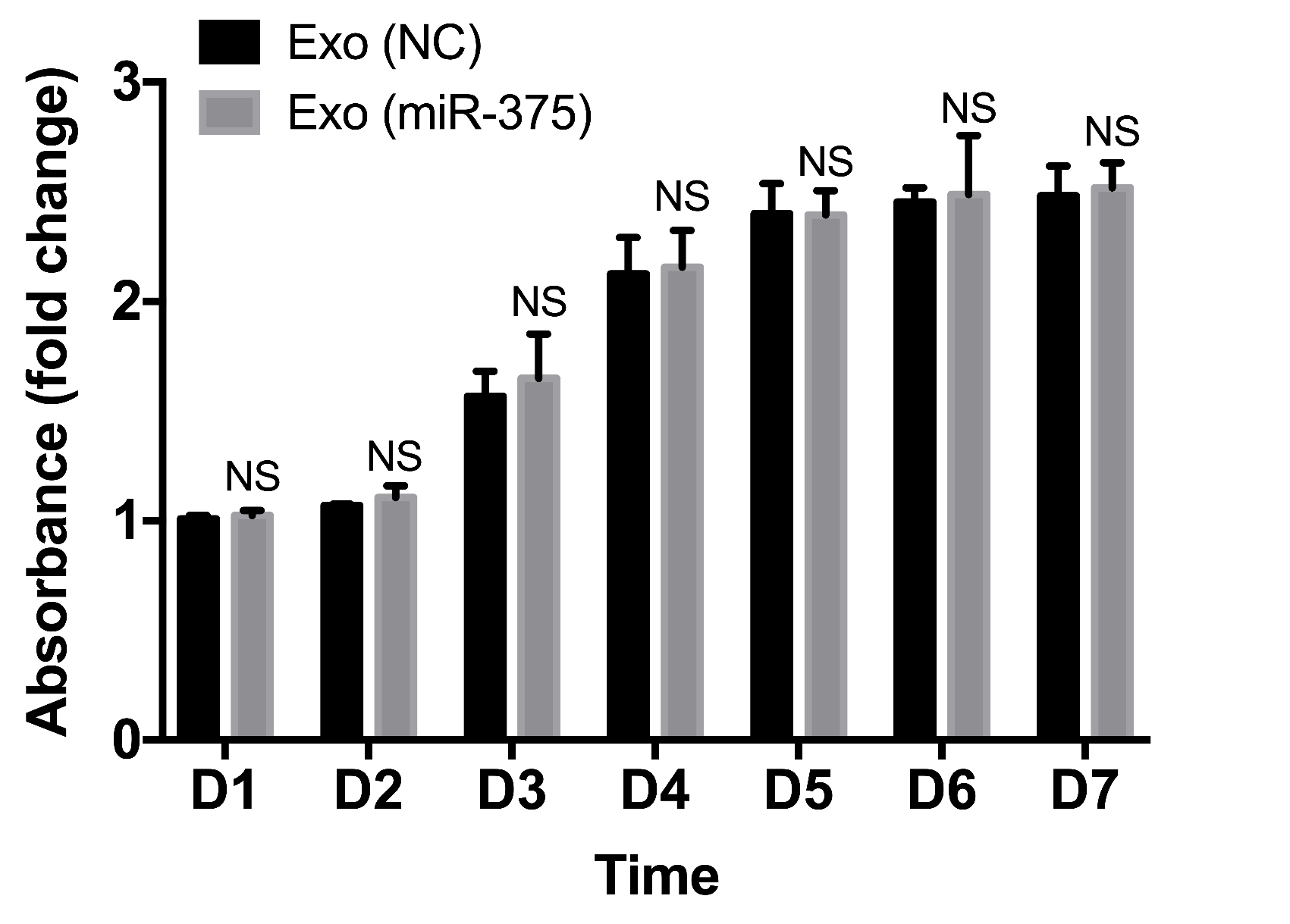


**Figure S2.** Exo (miR-375) made no effect on the proliferation of hBMSCs. Exo (miR-375) at 50 μg/ml was delivered into hBMSCs, and Exo (NC) at the same concentration was used as control. The growth ability of hBMSCs was measured with CCK-8 kit for 7 days. NS: not significant. hBMSCs, human bone marrow mesenchymal stem cells.


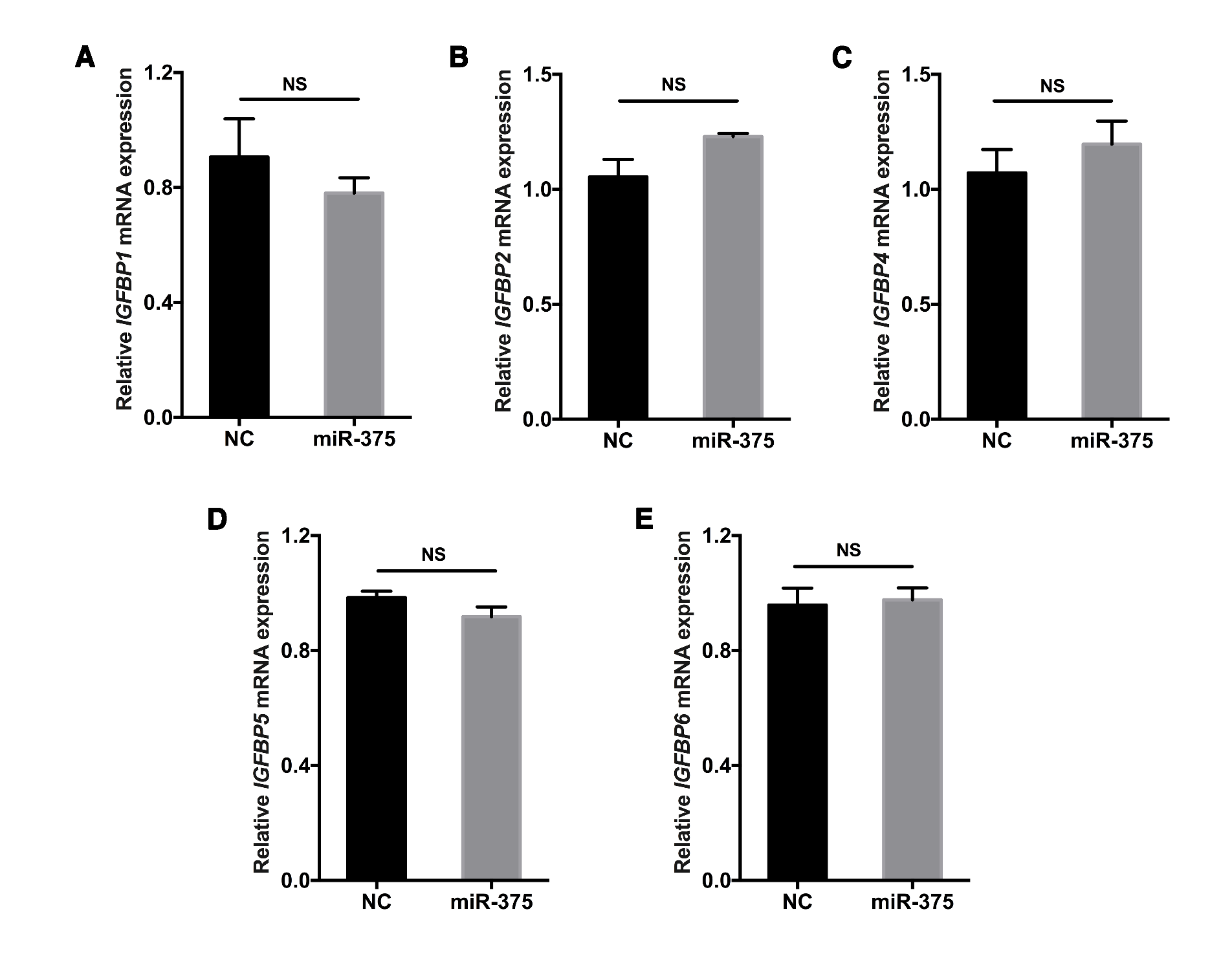


**Figure S3.** miR-375 has no effect on other members of IGFBP family. (A-E) hBMSCs were transfected with lentivirus overexpressing miR-375, and NC was used as the control vector. Relative mRNA levels of *IGFBP1*, *IGFBP2*, *IGFBP4*, *IGFBP5* and *IGFBP6* measured by qRT-PCR in the miR-375 and NC groups. *GAPDH* was used for normalization. Data are represented as mean ± SD; n = 3; NS: not significant. IGFBP1, insulin-like growth factor binding protein 1; IGFBP2, insulin-like growth factor binding protein 2; IGFBP4, insulin-like growth factor binding protein 4; IGFBP5, insulin-like growth factor binding protein 5; IGFBP6, insulin-like growth factor binding protein 6.


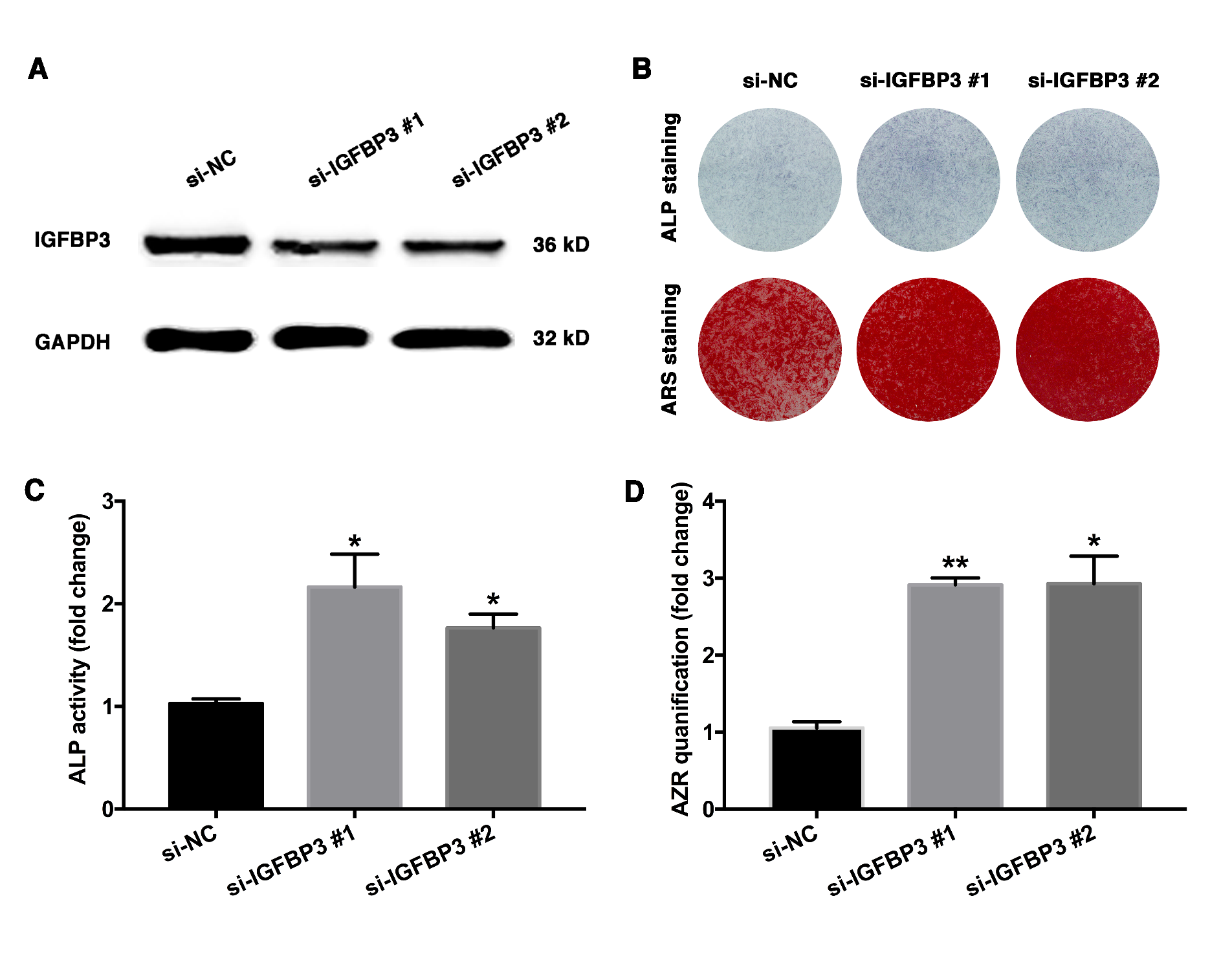


**Figure S4.** IGFBP3 inhibited the osteogenic differentiation of hBMSCs. hBMSCs were transfected with IGFBP3 siRNA (si-IGFBP3 #1, si-IGFBP3 #2) or the control vector (si-NC). (A) Western blotting showing IGFBP3 expression 48 h after transfection in the si-IGFBP3 #1, si-IGFBP3 #2, and si-NC groups. GAPDH was used as the internal control. (B) ALP staining on day 7 and ARS staining on day 14 after osteogenic induction in the si-IGFBP3 #1, si-IGFBP3 #2, and si-NC groups. (C, D) ALP activity on day 7 and ARS mineralization assay on day 14 after osteogenic induction. Data are represented as mean ± SD; n = 3; **P* < 0.05; ***P* < 0.01; NS: not significant.


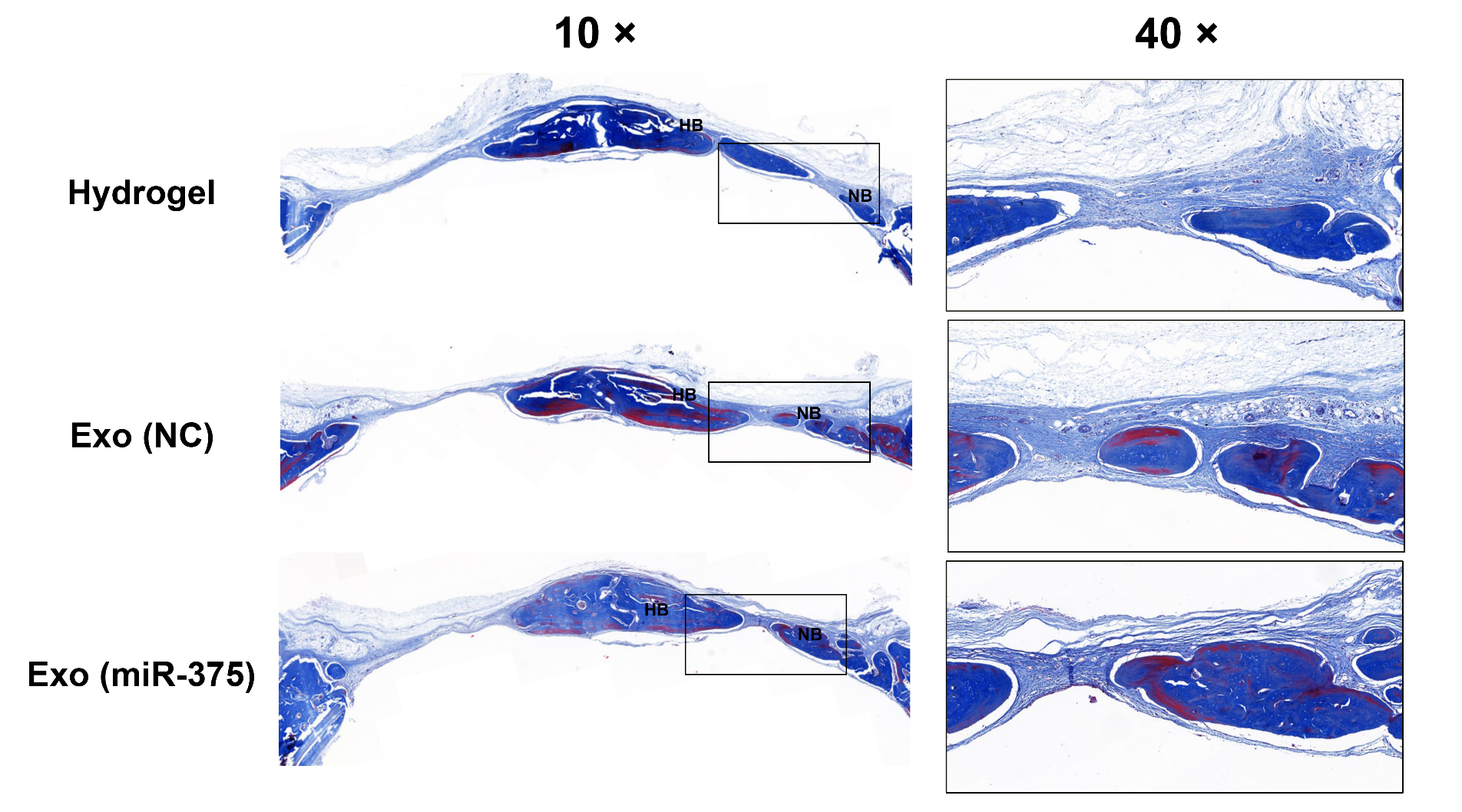


**Figure S5.** Masson staining of the newly formed bone 8 weeks after operation. Defects on the left side were left as the blank group without any treatment, and defects on the right side were treated with hydrogel, hydrogel loaded with Exo (NC), and hydrogel loaded with Exo (miR-375), and the magnification was respectively 10× and 40×. HB, host bone; NB, new bone.
